# Oncogenic KPNA2 Serves as a Biomarker and Immune Infiltration in Patients With HPV Positive Tongue Squamous Cell Carcinoma

**DOI:** 10.3389/fonc.2022.847793

**Published:** 2022-07-04

**Authors:** Li Gao, Ying Li, Cheng Yu, Dong-Xu Liu, Ke-Han Wu, Zhi-Li Wei, Ming-Yue Liu, Lei Yu

**Affiliations:** ^1^ Department of Oral and Maxillofacial Surgery, The Second Affiliated Hospital of Harbin Medical University, Harbin, China; ^2^ Department of stomatology, Daqing Oilfield General Hospital, Daqing, China; ^3^ Department of Colorectal Surgery, The Second Affiliated Hospital of Harbin Medical University, Harbin, China

**Keywords:** KPNA2, biomarker, immune infiltration, HPV, tongue squamous cell carcinoma

## Abstract

Human tongue squamous cell carcinoma (TSCC), the most prevalent type of oral cancer, is associated with human papillomavirus (HPV) infection. Our previous work showed Karyopherin α2 (KPNA2), as an oncogene of TSCC, by relegating the p53/autophagy signaling pathway. Nevertheless, the significance of KPNA2 in TSCC pathogenesis has not been established. KPNA2 levels were evaluated *via* the TCGA database, and its effects on survival outcomes were assessed by LASSO, Kaplan‐Meier, and COX regression analyses. CIBERSORT and ESTIMATE investigated the relationships between KPNA2 and immune infiltration. At the same time, KPNA2 and HPV infection was analyzed by immunohistochemistry. In addition, the association between downstream molecular regulation pathways and KPNA2 levels was determined by GO, GSEA, and WGCNA. In TSCC, KPNA2 levels were associated with clinical prognosis and tumor grade. Moreover, KPNA2 may be involved in cancer cell differentiation and facilitates tumor-related genes and signaling pathways, such as Cell Cycle, Mitotic G1 phase, G1/S transition, DNA Repair, and Transcriptional Regulation TP53 signaling pathways. Nevertheless, regulatory B cells, follicular helper B cells, and immune and stromal scores between low- and high-KPNA2 expression groups were insignificant. These results imply that KPNA2 is highly involved in tumor grade and prognosis of TSCC. KPNA2 levels correct with HPV 16 markedly regulated cell differentiation, several oncogenes, and cancer‐related pathways.

## Introduction

Tongue squamous cell carcinoma (TSCC), the most common form of head and neck squamous cell carcinoma (HNSCC), is associated with more than 500,000 cases annually. TSCC has high invasive behavior, local recurrence and high occult metastasis rate, and poor prognostic outcomes. Its mortality rate within five years is about 50% (1). TSCC account for more than 40% of all oral cancers. Patients with TSCC often suffer from severe oral dysfunction and face oral function reconstruction after surgical therapy (2). Although much progress in radiotherapy, chemotherapy, and surgery has been made in treating TSCC relative survival rate for TSCC patients is not markedly increased. The main risk factors for TSCC are drinking, smoking, and human papillomavirus (HPV). More than 23% of patients with TSCC are infected with HPV (3). HPV infection is responsible for the development and progression of some TSCCs with a unique tumorigenesis mechanism (4–9).

HPV is a double-stranded circular DNA virus and belongs to the papillomaviridae family, it has specific tropism to squamous epithelium. So far, 202 different HPV types have been isolated (International HPV Reference Center; http://ki.se/en/labmed/international-hpv-reference-center). The types of HPV infecting mucosa are further divided into high-risk type and high-risk type (10). High-risk HPV types HPV-16 and -18 are linked to 90% of uterine cervical cancers (11). Although the mechanism of this action is not completely clear, the detection of sensitive and specific biomarkers related to HPV in human TSCC has been compared and clarified, among which HPV16, E6 and E7 have been studied and discussed most frequently (12). HPV16 plays a leading role in HPV16, 18, 31, 45, 33, 52, 58, 35, 59, 56, 51, 6, 39, 68, 82, 66, 70 and 73, and is injected into 46-63% of squamous cell carcinomas, followed by HPV18 (10-14%) (13). HPV-positive oropharyngeal squamous cell carcinoma(OPSCCs) seem to have a better prognosis than HPV-negative ones, as they are less likely to recur locally and are more radiosensitive (14, 15). In the past 20 years, many studies regarding HPV and OSCCs have been conducted. HPV has been discussed as a relevant predictive biomarker of OSCC (16). Anatomically, there is no specific information (oral or oropharyngeal) about the samples. So far, it cannot be determined whether HPV infection has any practical impact on oral carcinogenesis (17). HPV infection, according to Attner et al., may cause a higher incidence of tongue cancer in adolescents (18). In addition to reducing treatment costs and improving quality of life, Anders Högmo et al. showed that different treatment strategies might increase patient satisfaction (19). Therefore, it is imperative to find new prognostic biomarkers. Identifying predictive markers would enable optimization of treatment and reduction of sequelae (20).

Cancer cells can evade the host immune system, which is a hallmark of cancer (21). Tumor-infiltrating lymphocytes (TIL) and their spatial organization in the tumor microenvironment are crucial in cancer progression (22). A solid tumor is classified histologically according to its immune profile. These immune profiles include T cells, also called tumor-immune phenotypes: immune-inflamed, immune-excluded, and immune-desert (23). Regarding the prognostic role of TILs in OSCC, several immunohistochemical studies have demonstrated an association between TIL levels of TILs and a better outcome. High CD3+ TIL levels at invasive tumor margins were significantly correlated associated with an improved prognosis in patients with OSCC (24). For the first time, Giuseppe Troiano et al. show that the immunophenotype of OTSCC predicts relapse and poor outcomes. At the same time, Giuseppe Troiano et al. also pointed out that current tumor lymph node metastasis systems lack the capability to identify patients at high risk of early recurrence and poor prognosis for OTSCC as well as patients with invasive cancers can be more accurately stratified based on their tumor immunophenotype (25). However, whether and how HPV plays an effective regulatory role in changing the survival rate of human TSCC patients has not been clarified (26, 27).Therefore, we hypothesize that HPV infection causes the TSCC to acquire an immunoinflammatory phenotype, and immune cells infiltrate the tumor, improving patient outcomes.

However, the relationship between KPNA2 and HPV-positive TSCC has not been established, especially the regulatory role of KPNA2 in HPV-positive TSCC. In our study, the prevalence of HPV 16 positively correlates with KPNA2 expression in TSCC. In addition, Immune cell infiltration provides a new tool for TSCC therapeutic approaches. We explored the tumor immune microenvironment(TIME) in a homogeneous TSCC cohort and evaluated its effects on survival outcomes.

## Materials and Methods

### Collection and Preprocessing of Data

The data on tongue squamous cell carcinoma (TSCC) contained gene expression data (HTSeq-Counts) of 137 TSCC patients from the ““CGA-HNSC” “project, and the clinical data tables corresponding to the samples were retrieved from Genomic Data Commons (https://portal.gdc.cancer.gov/). (Note: we collected the data in TCGA with TSCC, which showed HPV-positive TSCC or HPV-negative TSCC patients.) 124 TSCC tissue samples and 13 paracancerous tissue samples were contained among them.

To ensure study accuracy, we removed probes with more than 50% expression values equal to 0—additionally, the voom function in the ““imma” “package normalized gene expression profiles.

### Extrapolation of Tumor-Infiltrating Immune Cell

CIBERSORT has a deconvolution algorithm that can quantify the extent of infiltration of 272 tumor-infiltrating immune cells (TIICs) in individual samples. The abundance of TIICs for each model in 124 TSCC tissues was assessed by R (version 3.6.1) based on the CIBERSORT algorithm. Moreover, cytotoxic activity scores (CYT) and tumor-infiltrating lymphocyte scores (TILs) were calculated by the amount of expression of the genes.

### Differentially expressed genes (DEGs) associated with KPNA2 expression

The “imma” “package calculated DEGs associated with KPNA2 expression. The threshold for filtering differential genes was set at |*logFC*| > 1and *P* < 0.05.

### Enrichment Analysis of KPNA2 Co-Expression Genes

Metascape (http://metascape.org) is an online tool that offers several functions, including gene enrichment and protein interaction network analyses. Enrichment analysis was performed on 100 co-expressed genes of KPNA2 in the Metascape database. If multiple terms of GO or pathway annotations were identified, the top 20 most statistically significant terms were selected for visualization.

### Protein-Protein Interaction Network Establishment and Hub Genes Identification

We utilized the Search Tool for the Retrieval of Interacting Genes (STRING) (https://www.string-db.org/) database to build a PPI network and set the interaction score to 0.85. Cytoscape v3.7.0 was utilized to promote PPI network readability based on interactions in the STRING database. In addition, genes that exhibited direct interactions with KPNA2 were considered central hub genes.

### Patients and Samples

Tissue samples were resected from 30 TSCC patients who had been subjected to surgical procedures at the Department of Oral and Maxillofacial Surgery, The Second Affiliated Hospital of Harbin Medical University (Harbin, China) between April 2018 and March 2021. Tumor stages were determined under the Union for International Cancer Control TNM Classification of Malignant Tumors 7^th^ Edition, while histological grades were evaluated by the World Health Organization criteria for TSCC. This study was permitted by the Institutional Review Board of Harbin Medical University (KY2021-120, Harbin, China).

### Immunohistochemistry

The TSCC samples were fixed in formalin (phosphate-buffered formalin, 10%) for 24 h at room temperature (RT), paraffin-embedded, and sliced into 4-µm thick sections. Immunohistochemical (IHC) staining was done *via* the avidin-biotin-peroxidase complex strategy. Briefly, deparaffinized samples were heated in 10 mM citrate buffer (pH 6.0) at 121°C for 20 min for epitope regeneration. Incubation of sections was done in 0.3% hydrogen peroxide (in distilled water) at RT for 5 min to block endogenous peroxidase activities. This stage was followed by overnight incubation at four °C in the presence of a specific mouse monoclonal antibody to anti-human p16 and KPNA2. Sections were washed using TBS+Tween-20 (TBS-T; Sigma-Aldrich; Merck KGaA), overlaid with biotinylated anti-mouse antibodies at RT for 60 min, washed using TBS-T, and labeled with a streptavidin-peroxidase complex. Then, sections were stained with hematoxylin at RT for 10 s, dehydrated in graded ethanol (75, 95, 100, and 100%) for 5 minutes each at RT, treated with xylene, and then enclosed in synthetic resin. Observation of sections at ×40 and ×100 was done by a light microscope (Nikon Corporation). Positive p16 protein expressions were denoted as when >70% of tumor cells exhibited intense, diffuse nuclear and cytoplasmic staining.

### Statistical Analysis

Maximally Selected Log-rank Statistic was used as the sample classification. K-M analysis and plotting of heatmaps were applied with the “survival” “package and “heatmap” “package, respectively. Data from two groups were tested using analysis of variance, and the Kruskal-Wallis test was used to identify significance between three or more groups of data (* p ≤ 0.05; ** p ≤ 0.01; *** p ≤ 0.001; **** p ≤ 0.0001). Pearson correlation analyses determined the correlation between two variables. R (version 3.6.1) was used for our studies. Furthermore, when we assessed the significance of the results using *P*, *P* < 0.05 denoted matter.

## Results

### Diagnostic Value of KPNA2 in TSCC and the Association Between its Levels and Clinic-Pathological Characteristics

**Figure 1:**
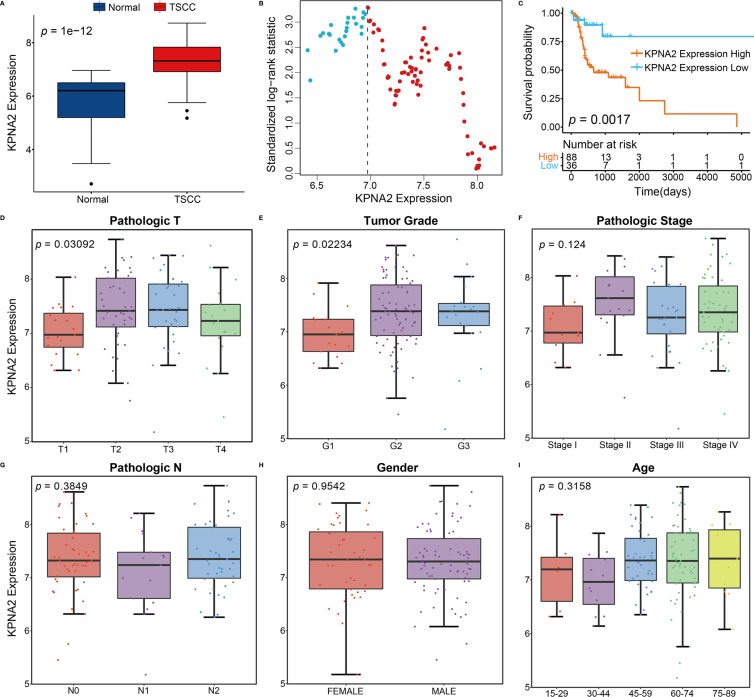
Association between KPNA2 expression and clinicopathological features of TSCC and diagnostic significance of KPNA2. **(A)** Cancer status. **(B)** Scatter plots show normalized log-rank statistical values for each respective cut-off of KPNA2 expression values. The vertical dashed line marks the best cut-off value with the most significant standard logarithmic rank statistic. **(C)** Kaplan-Meier plot for overall survival outcomes of TCGA-TSCC patients categorized based on KPNA2 expression. **(D)** Tumor invasion depth. **(E)** Tumor grade. **(F)** Stage. **(G)** Lymph node metastasis. **(H)** Gender. **(I)** Age.

In the TCGA cohort, the expression profile of KPNA2 was significantly different between 13 normal tissues and 124 TSCC tissue samples and showed a trend of high expression in TSCC tissues (**Figure 1A**). Next, 124 TSCC samples were assigned into high and low expression groups based on KPNA2 levels by maximally selected log-rank statistics (**Figure 1B**). As shown in **Figure 1C**, the KPNA2 low expression patient group’s survival rates were significantly higher than that of the high expression group (log-rank *P* = 0.0017). The association between KPNA2 levels and clinic-pathological characteristics was explored by collating the clinical features of TCGA-TSCC. As shown in **Figures 1D–I**, elevated KPNA2 levels markedly correlated with tumor invasion depth (*P* = 0.03092) and histological grade (*P* = 0.02234), however, no significant correlation was observed with lymph node metastasis (*P* = 0.3849), gender (*P* = 0.9542) and age (*P* = 0.3158). Moreover, as shown in **Table 1**, KPNA2 levels were significantly correlated with stage. Hence, TSCC was chosen as the primary target to investigate the potential role of KPNA2.

**Table 1:** Relationship between KPNA2 expression and clinical characteristics of TSCC patients.

	All cases	KPNA2		
		Low expression	High expression	*P*
**Gender**				
Female	44	16(36%)	28(64%)	0.2597
Male	80	20(25%)	60(75%)	
**Age**				
≥57.8	74	22(30%)	52(70%)	0.9948
<57.8	50	14(28%)	36(72%)	
**pT status**				
1	21	11(52%)	10(48%)	**0.02892**
2	43	9(21%)	34(79%)	
3	33	6(18%)	27(82%)	
4	19	5(26%)	14(74%)	
**pN status**				
0	48	12(25%)	36(75%)	0.3065
1	17	7(41%)	10(59%)	
2	45	10(22%)	35(78%)	
**Stage**				
I	13	7(54%)	6(46%)	**0.0498**
II	19	2(11%)	17(89%)	
III	29	8(28%)	21(72%)	
IV	51	12(24%)	39(76%)	
**Tumor grade**				
1	17	9(53%)	8(47%)	**0.04655**
2	85	23(27%)	62(73%)	
3	22	4(18%)	18(82%)	

pT, posttreatment tumor category; pN, lymph node status; Stage, AJCC prognostic stage grouping; Tumor grade, Tumor grade is a way of classifying tumors based on certain features of their cells.

Grade 1. The tumor cells look the most like normal tissue and are slow-growing (well-differentiated).

Grade 2. The tumor cells fall somewhere in between grade 1 and grade 3 (moderately-differentiated).

Grade 3. The tumor cells look very abnormal and are fast-growing (poorly-differentiated).

Bolded p-values were two-sided with significance defined as a p-value < 0.05.

### Relationship Between Immune Infiltrate Landscape and KPNA2 Expression

**Figure 2:**
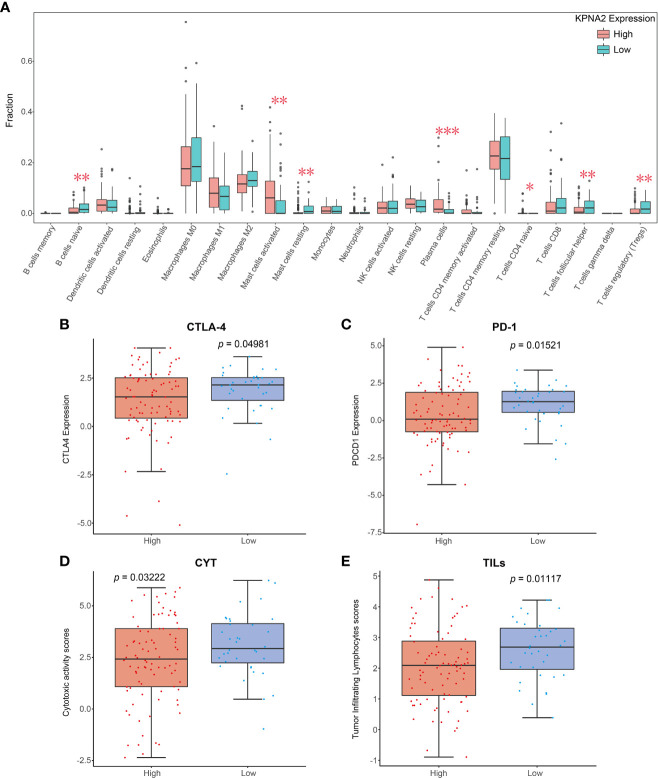
Relationship between KPNA2 levels and tumor immune landscape of TSCC. **(A)** Relationship between KPNA2 level and the proportion of 22 TIICs in TSCC tissues. **(B–E)** Box plots show different immune checkpoints between KPNA2 low- and high-expression cases. PD-1 (PDCD1), programmed cell death 1; CYT, cytotoxic activity; TILs, tumor-infiltrating lymphocytes; CTLA-4, cytotoxic T-lymphocyte associated protein 4. *P < 0.05, **P < 0.01 or ***P < 0.001, relative to High group.

To explore the mechanism by which KPNA2 participates in the pathological progression of TSCC, we evaluated the association between KPNA2 levels and 22 TIICs by the CIBERSORT algorithm based on expression profiles of TCGA. In TSCC tumor tissues, activated mast cells (*P* = 0.0044), plasma cells (*P* = 0.0005) and naive T cells CD4 (*P* = 0.0474) were highly infiltrated in high KPNA2 group, while naive B cells (*P* = 0.0080), resting mast cells (*P* = 0.0021), helper T cells follicular (*P* = 0.0164) and regulatory T cells (*P* = 0.0010) were all significantly enriched in the low KPNA2 group (**Figure 2A**). Immunotherapy has become an established mainstay of anticancer therapy to improve outcomes for patients with cancer. Therefore, the levels of common immune checkpoints in low and high KPNA2 groups were further examined in this study. As shown in **Figures 2B–E**, patients in the low KPNA2 group were markedly associated with higher levels of the cytotoxic T-lymphocyte-associated protein 4 (CTLA-4), program death-1 (PD-1), cytotoxic activity (CYT), tumor-infiltrating lymphocytes (TILs).

### Identification of Co-Expression Genes with KPNA2

**Figure 3:**
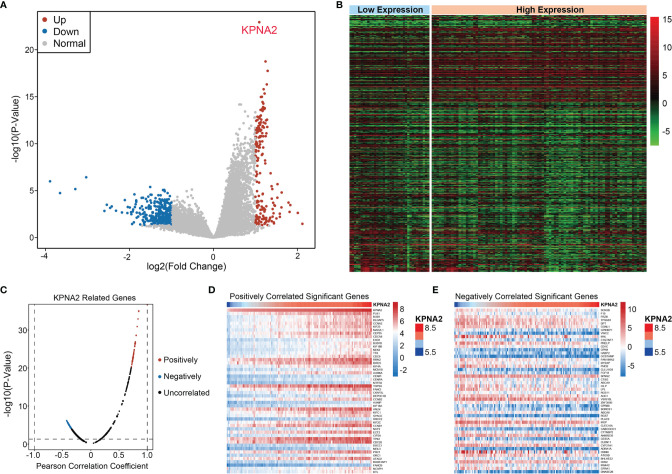
Identification of KPNA2 co-expression gene. **(A)** Volcano plot of DEGs associated with KPNA2 expression. Red denotes elevated DEGs, blue denotes suppressed DEGs, while gray denotes non-differential genes. **(B)** Heat map of DEGs. **(C)** Pearson test was used to assess the association between KPNA2 and DEGs. **(D, E)** The heat map shows genes positively and negatively correlated with KPNA2 (Top 50).

First, the “imma” “R package was utilized to derive 561 DEGs associated with KPNA2 expression, which contained 190 elevated DEGs and 371 suppressed DEGs (**Figures 3A, B**). Next, among these DEGs, the co-expression genes of KPNA2 were calculated by Pearson’s correlation analysis (**Figure 3C**). We selected genes with positive and negative correlation coefficients in the top 50, respectively, as co-expression genes (**Figures 3D, E**).

### Enrichments of Co-Expression Genes

**Figure 4:**
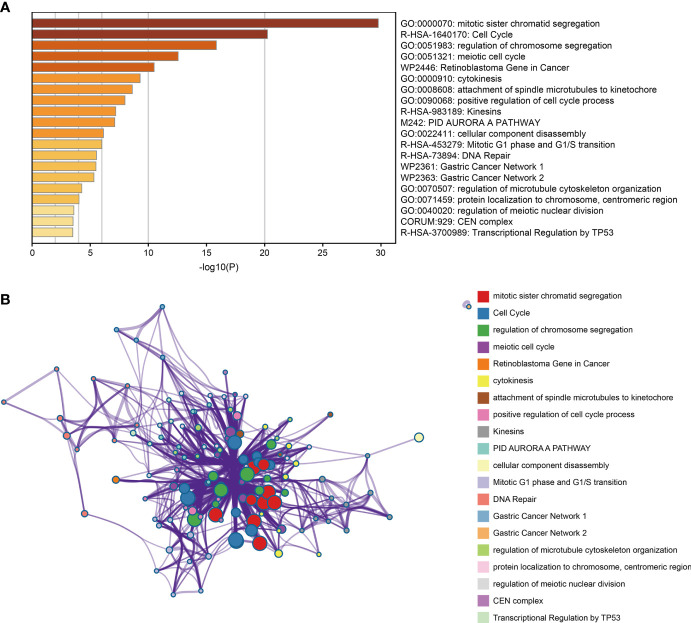
Functional enrichment analysis of co-expression gene. **(A)** Bar plot of the significantly top 20 terms enriched. **(B)** Network relationship plot between terms and terms.

Enrichment analysis of 100 co-expression genes was performed using the metascape database. These genes were highly enriched in several biological processes, such as mitotic sister chromatid segregation, chromosome segregation regulation, meiotic cell cycle, and positive regulation of cell cycle process. They were also mainly enriched in cell cycle, kinesins, and transcriptional regulation by TP53 pathways (**Figures 4A, B**).

### Protein-Protein Interaction Network and Hub Genes

**Figure 5:**
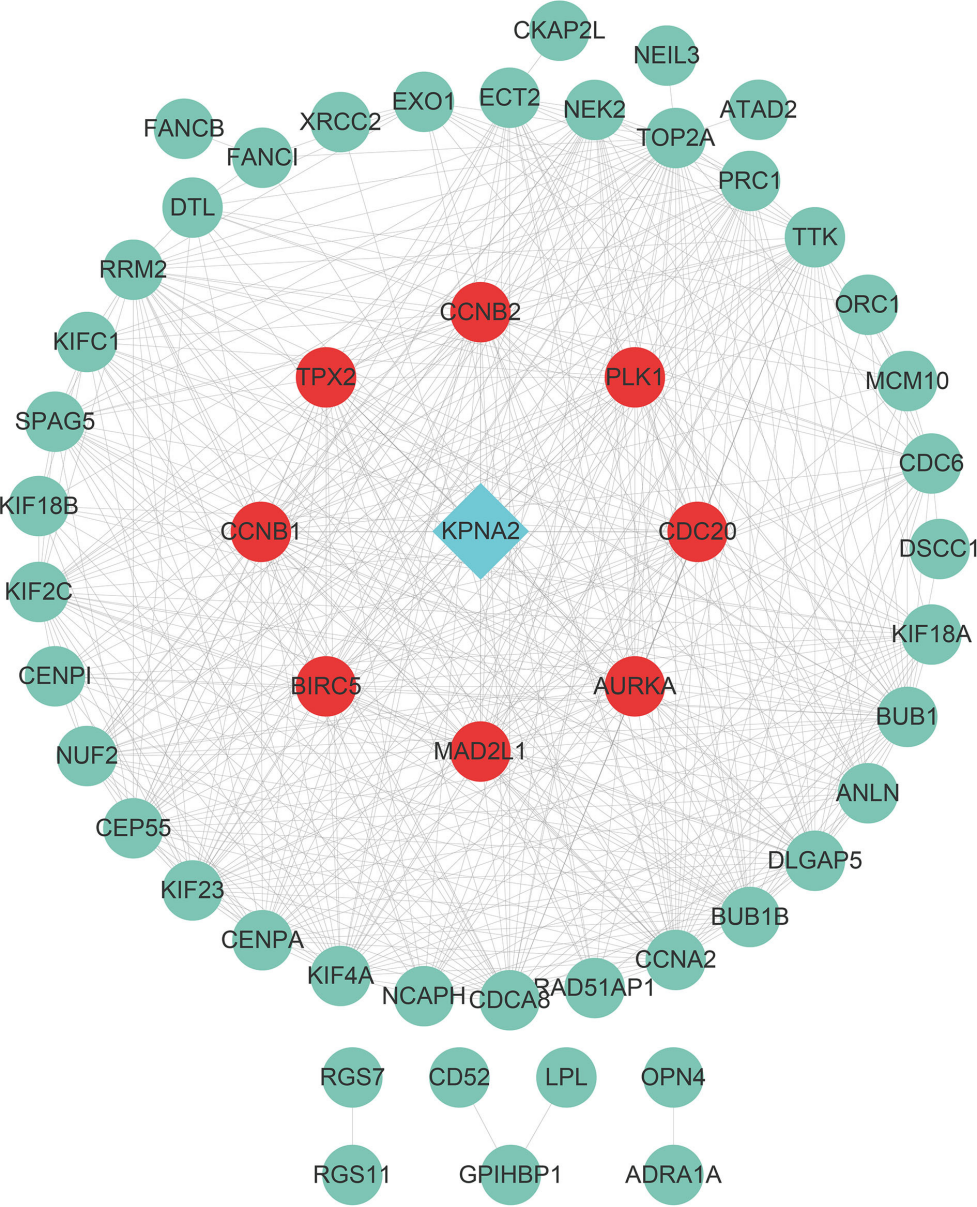
PPI network constructed from co-expression gene. Genes with red dots were directly associated with KPNA2 and were considered hub genes.

**Figure 6:**
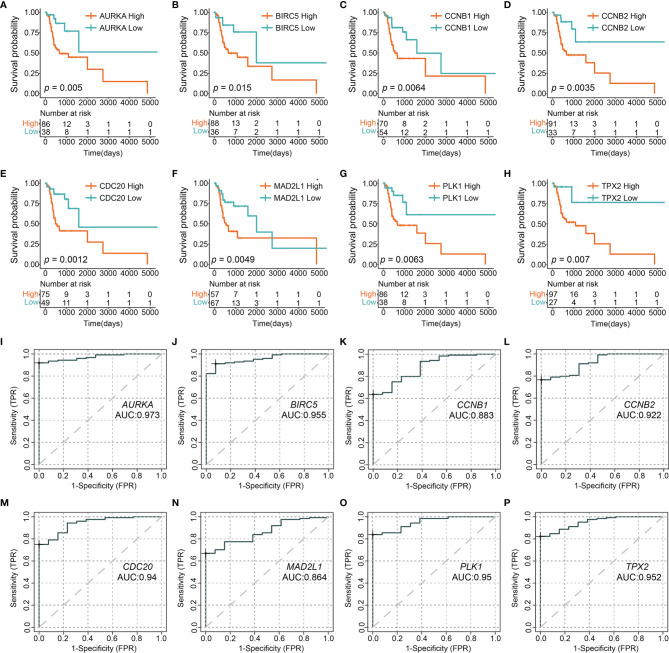
Kaplan–Meier survival analysis **(A–H)** and ROC curve **(I–P)** of eight hub genes of KPNA2.

A STRING database developed a PPI network to show intrinsic correlations between KPNA2 co-expression genes (**Figure 5**). Eight genes (AURKA, BIRC5, CCNB1, CCNB2, CDC20, MAD2L1, PLK1, and TPX2) were directly related to KPNA2 and were identified as central hub genes. Subsequently, we grouped the expression of the eight hub genes by Maximally Selected Log-rank Statistic and found that the eight hub genes with a low expression all had better survival than those with high expression (**Figures 6A–H**). Furthermore, the area under the curve (AUC) of these 8-hub gene receiver operating characteristic (ROC) curves all exceeded 0.85 (**Figures 6I–P**).

### The Relationship Between the KPNA2 mRNA Expression Level in Patients With Different HPV Status-TSCC and Their Prognosis Was Verified

**Figure 7:**
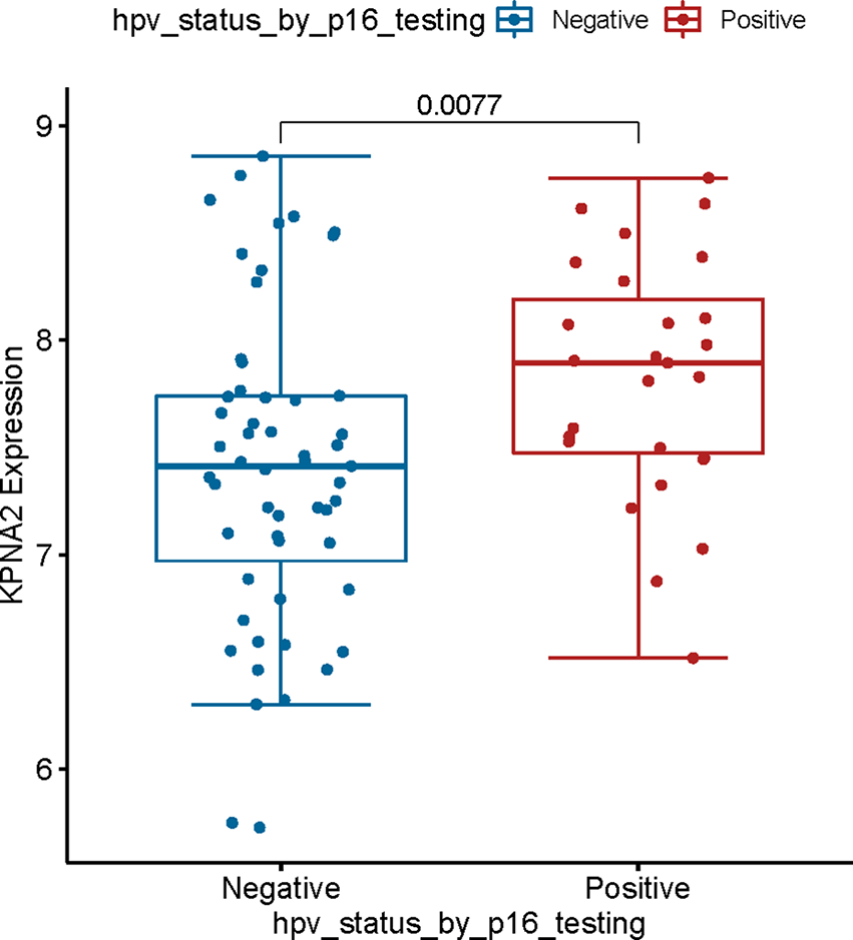
Validation of the relationship between KPNA2 expression and clinical information. KPNA2 was up-regulated in patients with HPV-positive TSCC patients.

To our knowledge, HPV plays a significant role in TSCC progress (28).On the other hand, much evidence shows that HPV16, a biomarker of HPV, is an upstream agent that can infect genes in TSCC progress (29). However, the relationship between HPV16 and KPNA2 in OSCC is unclear. To detect this hypothesis, we used TCGA-OSCC datasets with HPV infection information. Our results showed that HPV16 positively correlates with KPNA2 expression in TSCC (**Figure 7**). The abovementioned effects confirmed that KPNA2 was up-regulated in HPV-positive TSCC, unlike in HPV-negative TSCC.

**Figure 8:**
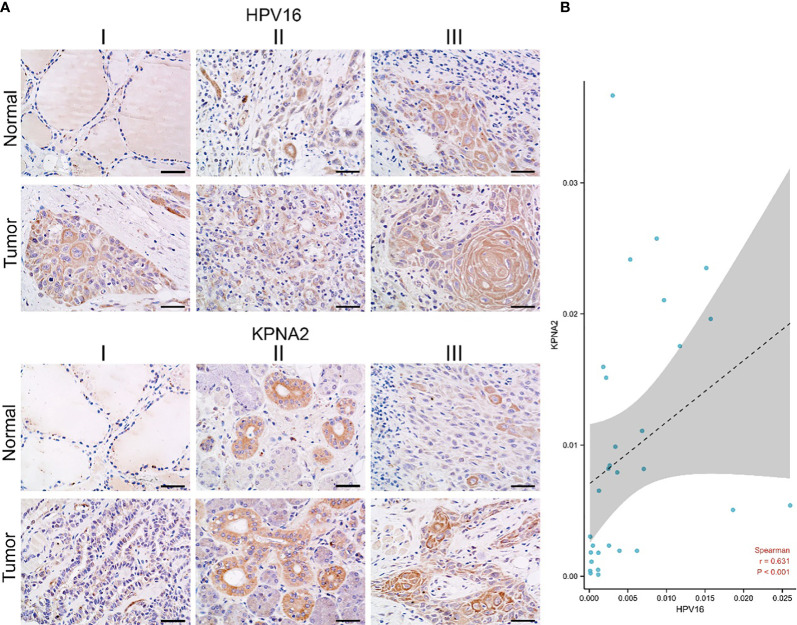
The correlation of KPNA2 and HPV16 in TSCC patients. **(A)** Immunohistochemistry, **(B)** statistical data between KPNA2 and HPV16.

To investigate the relationship between KPNA2 and HPV16 in TSCC patients. Immunohistochemistry was employed to test the expression of KPNA2 and HPV in TSCC tissues. In our data, KPNA2 is up-regulated in TSCC tissue, while HPV16 is also up-regulated in TSCC tissues (**Figure 8A**). And also, KPNA2 and HPV16 positively correlated in TSCC patients (**Figure 8B**).

## Discussion

KPNA2, a nuclear export protein involved in tumorigenesis, is a potential target in oncology (30).In our previous work, we found KPNA2 is up-regulated in TSCC. And also, knockdown KPNA2 can regulate TSCC cell growth and induce apoptosis by regulating the p53/autophagy signaling pathway (31, 32). However, other mechanisms and upstream influencing factors for KPNA2 in TSCC progress have not been established. We want to explore the association between KPNA2 and HPV in TSCC, especially downstream mechanisms.

There is no significant difference in the current treatment options for HPV+ TSCC compared with HPV-only TSCC. TSCC patients may be left with disfigurement and masticatory dysfunction after surgery. However, platinum-based chemoradiation is linked to debilitating health complications, including dysphagia, xerostomia, and ototoxicity (33). HPV+ TSCC patients may experience high incidence rates. In addition to being younger than HPV-positive patients, they generally share a declining quality of life after receiving cancer treatment (34). In addition, HPV+ TSCC patients seem to respond favorably to treatment, so de-escalation strategies are becoming increasingly popular for this patient group. Our study evaluated the predictive landscape of carbonic anhydrase in TSCC. KPNA2 levels were significantly correlated with survival outcomes for TSCC patients (**Table 1**).

Moreover, there was a significant correlation between KPNA2 levels and prognostic outcomes for TSCC patients. KPNA2 has been shown to mediate the survival outcomes for oral squamous cell carcinoma patients. Therefore, it is a potential independent prognostic marker. Moreover, KPNA2 levels were correlated with tumor grade. These findings imply that KPNA2 is a possible diagnostic and prognostic factor for TSCC.

On the other hand, GSEA revealed that KPNA2 levels were negatively associated with mitotic sister chromatid segregation and the Cell Cycle signaling pathway. Elevated KPNA2 levels markedly suppressed the infiltration levels of naive B cell, activated Mast cells, Mast cell resting, plasma cells, T cells follicular helper, and Tregs, which could be attributed to the suppressed B cell proliferation and migration. KPNA2 can induce immunogenic cell death by radiation (35). B cell regulation could also be attributed to expressions of KPNA2‐specific protein epitopes in TSCC.

At the molecular level, HPV + TSCC is characterized by wild-type p53, a major tumor suppressor protein, usually mutated in HPV -TSCC and most cancers from other anatomical sites (36). Our previous studies found that knocking down KPNA2 inhibited cell migration, autophagy, and cisplatin resistance by decreasing p53 translocation nucleocytoplasmic (32). The tumor-inhibiting properties of p53 in HPV + TSCC are affected by viral oncoprotein E6 in two distinct ways. The HPV E6 ubiquitin ligase, E6AP, promotes the degradation of wild-type p53 by the proteasome (36–39). HPV E6 binds to P300 to block the acetylation of p53, thus rendering p53 less stable and less active as a transcription factor (40–45).

As a result of infection with HPV, HPV-positive tumors typically have an increased CRT response that activates the immune system and results in a high proportion of immune cells infiltrating the tumor. Therefore, it will exert a more powerful antitumor effect (46). Those tumors which are HPV-negative, however, are believed to be immune-suppressed. Therefore, HNSCC immunotherapy efficacy may be negatively impacted by HPV infection (47, 48). According to a meta-analysis of 11 studies published in 2020, HNSCC patients with HPV were 1.29 times more likely to respond to immunotherapy than HNSCC patients without HPV (risk ratio 1.29; 95% CI = 0.85-1.96; I2 = 0), and it is twice as likely that they will survive (11.5 months vs. 6.3 months) (49). Hence, better immunotherapy efficacy may be associated with a better prognosis of HPV-related TSCC. Since risk stratification based on tumor size, lymph nodes and distant metastasis (TNM stage) and histological grade alone are not enough to predict the prognosis of TSCC patients, additional prognostic biomarkers are urgently needed (50). As an endpoint of our study, we found that HPV16 positively correlates with KPNA2 expression in TSCC (**Figure 7**). As well as, HPV16 E6 also positively correlates with the presentation of KPNA2 in TSCC patients (**Figure 8**).

Through COX regression, KPNA2 was established to be highly related to tumor grade in patients with tongue carcinoma. Therefore, we evaluated tumor grade co‐expressed genes in low and high expression groups *via* WGCNA. It was established that genes associated with tumor grade were enriched in cell differentiation and negative regulation of cell proliferation. In sinonasal papilloma, KPNA2 levels were associated with cancer cell differentiation and proliferation and showed potential cancer recurrence biomarkers. In pancreatic ductal adenocarcinoma, KPNA2 expression in adenocarcinoma was significantly suppressed in well‐differentiated cases. KPNA2 probably affected TSCC grade by modulating neoplastic cell differentiation and proliferation. Moreover, hug genes, including CCNB2, PLK1, CDC20, AURKA, MAD2L1, BIRC5, CCNB1, and TPX2, participated. In TSCC, CCNB2 and CDC20 were novel therapies for TSCC Patients with High-Grade Tumors (51). BIRC5 was inversely associated with the characterization of antigen processing machinery and survival expression in tonsillar squamous cell carcinoma (52). CCNB1 promotes cell proliferation and tumorigenesis *via* p53-related pathways in OSCC (53). Based on the findings, we postulated that during cancer cell differentiation, KPNA2 activates various co‐expressed genes that affect the differentiation of TSCC.

In conclusion, elevated KPNA2 levels in TSCC are associated with poor prognostic outcomes and tumor grades. Activations of tumor-related pathways and cell differentiation‐associated genes are vital in these biological processes correct with HPV16. It will be possible to control the incidence rate of HPV related tongue squamous cell carcinoma, improve the prognosis, provide reference for patients’ treatment options, and improve the survival rate and quality of life of patients.It is indispensable to perform cytological and animal experiments to confirm these conclusions.

## Data Availability Statement

The original contributions presented in the study are included in the article/supplementary material. Further inquiries can be directed to the corresponding author.

## Ethics Statement

The studies involving human participants were reviewed and approved by Harbin Medical University (KY2021-120, Harbin, China). The patients/participants provided their written informed consent to participate in this study.

## Author Contributions

Conceptualization: LG and YL. Methodology: CY, D-X L and K-HW. Statistical analysis: Z-LW and M-YL. Investigation: LG, YL and D-X L. Writing: LG and YL. Supervision: LY. All authors have read and approved the final manuscript.

## Funding

Medical Wisdom Research Fund supported this research by the Heilongjiang Sunshine Health Foundation (H21L0802). Harbin Medical University Scientific Research Innovation Fund (no. S202110226059). Special funds for Hei Long Jiang Postdoctoral Foundation (LBH-Z18218). Health and Family Planning Commission of Heilongjiang province (2018032). Heilongjiang Academy of Medical Science (201703).

## Conflict of Interests

The authors declare that the research was conducted in the absence of any commercial or financial relationships that could be construed as a potential conflict of interest.

## Publisher’s Note

All claims expressed in this article are solely those of the authors and do not necessarily represent those of their affiliated organizations, or those of the publisher, the editors and the reviewers. Any product that may be evaluated in this article, or claim that may be made by its manufacturer, is not guaranteed or endorsed by the publisher.

## References

[1] Johnson DE Burtness B Leemans CR Lui VWY Bauman JE Grandis JR. Head and Neck Squamous Cell Carcinoma. Nat Rev Dis Primers (2020) 6(1):92. doi: 10.1038/s41572-020-00224-3 33243986 PMC7944998

[2] Rivera C Oliveira AK Costa RAP De Rossi T Paes Leme AF. Prognostic Biomarkers in Oral Squamous Cell Carcinoma: A Systematic Review. Oral Oncol (2017) 72:38–47. doi: 10.1016/j.oraloncology.2017.07.003 28797460

[3] Dayyani F Etzel CJ Liu M Ho CH Lippman SM Tsao AS. Meta-Analysis of the Impact of Human Papillomavirus (HPV) on Cancer Risk and Overall Survival in Head and Neck Squamous Cell Carcinomas (HNSCC). Head Neck Oncol (2010) 2:15. doi: 10.1186/1758-3284-2-15 20587061 PMC2908081

[4] Dahlgren L Dahlstrand HM Lindquist D Hogmo A Bjornestal L Lindholm J. Human Papillomavirus is More Common in Base of Tongue Than in Mobile Tongue Cancer and Is a Favorable Prognostic Factor in Base of Tongue Cancer Patients. Int J Cancer (2004) 112(6):1015–9. doi: 10.1002/ijc.20490 15386365

[5] Mork J Lie AK Glattre E Hallmans G Jellum E Koskela P. Human Papillomavirus Infection as a Risk Factor for Squamous-Cell Carcinoma of the Head and Neck. N Engl J Med (2001) 344(15):1125–31. doi: 10.1056/NEJM200104123441503 11297703

[6] Gillison ML Shah KV. Human Papillomavirus-Associated Head and Neck Squamous Cell Carcinoma: Mounting Evidence for an Etiologic Role for Human Papillomavirus in a Subset of Head and Neck Cancers. Curr Opin Oncol (2001) 13(3):183–8. doi: 10.1097/00001622-200105000-00009 11307062

[7] Alani RM Munger K. Human Papillomaviruses and Associated Malignancies. J Clin Oncol (1998) 16(1):330–7. doi: 10.1200/JCO.1998.16.1.330 9440761

[8] D'Souza G Kreimer AR Viscidi R Pawlita M Fakhry C Koch WM. Case-Control Study of Human Papillomavirus and Oropharyngeal Cancer. N Engl J Med (2007) 356(19):1944–56. doi: 10.1056/NEJMoa065497 17494927

[9] Smith EM Ritchie JM Summersgill KF Klussmann JP Lee JH Wang D. Age, Sexual Behavior and Human Papillomavirus Infection in Oral Cavity and Oropharyngeal Cancers. Int J Cancer (2004) 108(5):766–72. doi: 10.1002/ijc.11633 14696105

[10] zur Hausen H. Papillomaviruses and Cancer: From Basic Studies to Clinical Application. Nat Rev Cancer (2002) 2(5):342–50. doi: 10.1038/nrc798 12044010

[11] Mincione GP Taddei GL Wolovsky M Calzolari A Mincione F. Detection of Human Papillomavirus (HPV) DNA Type 6/11 in a Conjunctival Papilloma by *in Situ* Hybridization With Biotinylated Probes. Pathologica (1092) 1992:483–8:84. 1337200

[12] Gipson BJ Robbins HA Fakhry C D'Souza G. Sensitivity and Specificity of Oral HPV Detection for HPV-Positive Head and Neck Cancer. Oral Oncol (2018) 77:52–6. doi: 10.1016/j.oraloncology.2017.12.008 PMC5788034 29362127

[13] Duray A Descamps G Decaestecker C Remmelink M Sirtaine N Lechien J. Human Papillomavirus DNA Strongly Correlates With a Poorer Prognosis in Oral Cavity Carcinoma. Laryngoscope (2012) 122(7):1558–65. doi: 10.1002/lary.23298 22532307

[14] Schache AG Liloglou T Risk JM Filia A Jones TM Sheard J. Evaluation of Human Papilloma Virus Diagnostic Testing in Oropharyngeal Squamous Cell Carcinoma: Sensitivity, Specificity, and Prognostic Discrimination. Clin Cancer Res (2011) 17(19):6262–71. doi: 10.1158/1078-0432.CCR-11-0388 PMC3188400 21969383

[15] Bishop JA Ma XJ Wang H Luo Y Illei PB Begum S. Detection of Transcriptionally Active High-Risk HPV in Patients With Head and Neck Squamous Cell Carcinoma as Visualized by a Novel E6/E7 mRNA *in Situ* Hybridization Method. Am J Surg Pathol (2012) 36(12):1874–82. doi: 10.1097/PAS.0b013e318265fb2b PMC3500437 23060353

[16] Jemal A Siegel R Ward E Hao Y Xu J Thun MJ. Cancer Statistics, 2009. CA Cancer J Clin (2009) 59(4):225–49. doi: 10.3322/caac.20006 19474385

[17] Panzarella V Campisi G Giardina Y Maniscalco L Capra G Rodolico V. Low Frequency of Human Papillomavirus in Strictly Site-Coded Oral Squamous Cell Carcinomas, Using the Latest NHI/SEER-ICD Systems: A Pilot Observational Study and Critical Review. Cancers (Basel) (2021) 13(18). doi: 10.3390/cancers13184595 PMC8472531 34572821

[18] Attner P Du J Nasman A Hammarstedt L Ramqvist T Lindholm J. The Role of Human Papillomavirus in the Increased Incidence of Base of Tongue Cancer. Int J Cancer (2010) 126(12):2879–84. doi: 10.1002/ijc.24994 19856308

[19] Hogmo A Holmberg E Haugen Cange H Reizenstein J Wennerberg J Beran M. Base of Tongue Squamous Cell Carcinomas, Outcome Depending on Treatment Strategy and P16 Status. A Population-Based Study From the Swedish Head and Neck Cancer Register. Acta Oncol (2022) 61(4):433–40. doi: 10.1080/0284186X.2022.2027516 35081863

[20] Kano S Sakashita T Tsushima N Mizumachi T Nakazono A Suzuki T. Validation of the 8th Edition of the AJCC/UICC TNM Staging System for Tongue Squamous Cell Carcinoma. Int J Clin Oncol (2018) 23(5):844–50. doi: 10.1007/s10147-018-1276-5 29675646

[21] Hanahan D Weinberg RA. Hallmarks of Cancer: The Next Generation. Cell (2011) 144(5):646–74. doi: 10.1016/j.cell.2011.02.013 21376230

[22] Shaban M Khurram SA Fraz MM Alsubaie N Masood I Mushtaq S. A Novel Digital Score for Abundance of Tumour Infiltrating Lymphocytes Predicts Disease Free Survival in Oral Squamous Cell Carcinoma. Sci Rep (2019) 9(1):13341. doi: 10.1038/s41598-019-49710-z 31527658 PMC6746698

[23] Hegde PS Karanikas V Evers S. The Where, the When, and the How of Immune Monitoring for Cancer Immunotherapies in the Era of Checkpoint Inhibition. Clin Cancer Res (2016) 22(8):1865–74. doi: 10.1158/1078-0432.CCR-15-1507 27084740

[24] Zhou C Wu Y Jiang L Li Z Diao P Wang D. Density and Location of CD3(+) and CD8(+) Tumor-Infiltrating Lymphocytes Correlate With Prognosis of Oral Squamous Cell Carcinoma. J Oral Pathol Med (2018) 47(4):359–67. doi: 10.1111/jop.12698 29469989

[25] Troiano G Rubini C Togni L Caponio VCA Zhurakivska K Santarelli A. The Immune Phenotype of Tongue Squamous Cell Carcinoma Predicts Early Relapse and Poor Prognosis. Cancer Med (2020) 9(22):8333–44. doi: 10.1002/cam4.3440 PMC7666743 33047888

[26] Hubbers CU Akgul B. HPV and Cancer of the Oral Cavity. Virulence (2015) 6(3):244–8. doi: 10.1080/21505594.2014.999570 PMC4601238 25654476

[27] Gotz C Drecoll E Straub M Bissinger O Wolff KD Kolk A. Impact of HPV Infection on Oral Squamous Cell Carcinoma. Oncotarget (2016) 7(47):76704–12. doi: 10.18632/oncotarget.12501 PMC5363542 27732948

[28] Rautava J Syrjanen S. Biology of Human Papillomavirus Infections in Head and Neck Carcinogenesis. Head Neck Pathol (2012) 6 Suppl 1:S3–15. doi: 10.1007/s12105-012-0367-2 22782219 PMC3394166

[29] Kerishnan JP Gopinath SC Kai SB Tang TH Ng HL Rahman ZA. Detection of Human Papillomavirus 16-Specific IgG and IgM Antibodies in Patient Sera: A Potential Indicator of Oral Squamous Cell Carcinoma Risk Factor. Int J Med Sci (2016) 13(6):424–31. doi: 10.7150/ijms.14475 PMC4893556 27279791

[30] Sun Y Li W Li X Zheng H Qiu Y Yang H. Oncogenic Role of Karyopherin Alpha2 (KPNA2) in Human Tumors: A Pan-Cancer Analysis. Comput Biol Med (2021) 139:104955. doi: 10.1016/j.compbiomed.2021.104955 34735944

[31] Gao L Yu L Li CM Li Y Jia BL Zhang B. Karyopherin Alpha2 Induces Apoptosis in Tongue Squamous Cell Carcinoma CAL-27 Cells Through the P53 Pathway. Oncol Rep (2016) 35(6):3357–62. doi: 10.3892/or.2016.4750 27109484

[32] Lin F Gao L Su Z Cao X Zhan Y Li Y. Knockdown of KPNA2 Inhibits Autophagy in Oral Squamous Cell Carcinoma Cell Lines by Blocking P53 Nuclear Translocation. Oncol Rep (2018) 40(1):179–94. doi: 10.3892/or.2018.6451 PMC6059741 29781035

[33] Machtay M Moughan J Trotti A Garden AS Weber RS Cooper JS. Factors Associated With Severe Late Toxicity After Concurrent Chemoradiation for Locally Advanced Head and Neck Cancer: An RTOG Analysis. J Clin Oncol (2008) 26(21):3582–9. doi: 10.1200/JCO.2007.14.8841 PMC4911537 18559875

[34] Ang KK Harris J Wheeler R Weber R Rosenthal DI Nguyen-Tan PF. Human Papillomavirus and Survival of Patients With Oropharyngeal Cancer. N Engl J Med (2010) 363(1):24–35. doi: 10.1056/NEJMoa0912217 20530316 PMC2943767

[35] Song KH Jung SY Kang SM Kim MH Ahn J Hwang SG. Induction of Immunogenic Cell Death by Radiation-Upregulated Karyopherin Alpha 2 *In Vitro* . Eur J Cell Biol (2016) 95(6-7):219–27. doi: 10.1016/j.ejcb.2016.04.002 27107455

[36] Stransky N Egloff AM Tward AD Kostic AD Cibulskis K Sivachenko A. The Mutational Landscape of Head and Neck Squamous Cell Carcinoma. Science (2011) 333(6046):1157–60. doi: 10.1126/science.1208130 PMC3415217 21798893

[37] Scheffner M Werness BA Huibregtse JM Levine AJ Howley PM. The E6 Oncoprotein Encoded by Human Papillomavirus Types 16 and 18 Promotes the Degradation of P53. Cell (1990) 63(6):1129–36. doi: 10.1016/0092-8674(90)90409-8 2175676

[38] Scheffner M Huibregtse JM Vierstra RD Howley PM. The HPV-16 E6 and E6-AP Complex Functions as a Ubiquitin-Protein Ligase in the Ubiquitination of P53. Cell (1993) 75(3):495–505. doi: 10.1016/0092-8674(93)90384-3 8221889

[39] Huibregtse JM Scheffner M Howley PM. A Cellular Protein Mediates Association of P53 With the E6 Oncoprotein of Human Papillomavirus Types 16 or 18. EMBO J (1991) 10(13):4129–35. doi: 10.1002/j.1460-2075.1991.tb04990.x PMC453163 1661671

[40] Zimmermann H Degenkolbe R Bernard HU O'Connor MJ. The Human Papillomavirus Type 16 E6 Oncoprotein can Down-Regulate P53 Activity by Targeting the Transcriptional Coactivator CBP/P300. J Virol (1999) 73(8):6209–19. doi: 10.1128/JVI.73.8.6209-6219.1999 PMC112697 10400710

[41] Patel D Huang SM Baglia LA McCance DJ. The E6 Protein of Human Papillomavirus Type 16 Binds to and Inhibits Co-Activation by CBP and P300. EMBO J (1999) 18(18):5061–72. doi: 10.1093/emboj/18.18.5061 PMC1171577 10487758

[42] Thomas MC Chiang CM. E6 Oncoprotein Represses P53-Dependent Gene Activation *via* Inhibition of Protein Acetylation Independently of Inducing P53 Degradation. Mol Cell (2005) 17(2):251–64. doi: 10.1016/j.molcel.2004.12.016 15664194

[43] Ito A Kawaguchi Y Lai CH Kovacs JJ Higashimoto Y Appella E. MDM2-HDAC1-Mediated Deacetylation of P53 is Required for its Degradation. EMBO J (2002) 21(22):6236–45. doi: 10.1093/emboj/cdf616 PMC137207 12426395

[44] Li M Luo J Brooks CL Gu W. Acetylation of P53 Inhibits Its Ubiquitination by Mdm2. J Biol Chem (2002) 277(52):50607–11. doi: 10.1074/jbc.C200578200 12421820

[45] Xie X Piao L Bullock BN Smith A Su T Zhang M. Targeting HPV16 E6-P300 Interaction Reactivates P53 and Inhibits the Tumorigenicity of HPV-Positive Head and Neck Squamous Cell Carcinoma. Oncogene (2014) 33(8):1037–46. doi: 10.1038/onc.2013.25 PMC3912227 23474763

[46] Stanley M. HPV - Immune Response to Infection and Vaccination. Infect Agent Cancer (2010) 5:19. doi: 10.1186/1750-9378-5-19 20961432 PMC3161350

[47] Whiteside TL. Immune Responses to Cancer: Are They Potential Biomarkers of Prognosis? Front Oncol (2013) 3:107. doi: 10.3389/fonc.2013.00107 23730621 PMC3656353

[48] Nguyen N Bellile E Thomas D McHugh J Rozek L Virani S. Tumor Infiltrating Lymphocytes and Survival in Patients With Head and Neck Squamous Cell Carcinoma. Head Neck (2016) 38(7):1074–84. doi: 10.1002/hed.24406 PMC4900934 26879675

[49] Galvis MM Borges GA Oliveira TB Toledo IP Castilho RM Guerra ENS. Immunotherapy Improves Efficacy and Safety of Patients With HPV Positive and Negative Head and Neck Cancer: A Systematic Review and Meta-Analysis. Crit Rev Oncol Hematol (2020) 150:102966. doi: 10.1016/j.critrevonc.2020.102966 32371338

[50] Lothaire P de Azambuja E Dequanter D Lalami Y Sotiriou C Andry G. Molecular Markers of Head and Neck Squamous Cell Carcinoma: Promising Signs in Need of Prospective Evaluation. Head Neck (2006) 28(3):256–69. doi: 10.1002/hed.20326 16284973

[51] Lin H Chen L Li W Chen Z. Novel Therapies for Tongue Squamous Cell Carcinoma Patients With High-Grade Tumors. Life (Basel) (2021) 11(8):796–802. doi: 10.3390/life11080813 34440557 PMC8398384

[52] Weinman EC Roche PC Kasperbauer JL Cha SS Sargent DJ Cheville J. Characterization of Antigen Processing Machinery and Survivin Expression in Tonsillar Squamous Cell Carcinoma. Cancer (2003) 97(9):2203–11. doi: 10.1002/cncr.11311 12712472

[53] Qiu L Liu H Wang S Dai XH Shang JW Lian XL. FKBP11 Promotes Cell Proliferation and Tumorigenesis *via* P53-Related Pathways in Oral Squamous Cell Carcinoma. Biochem Biophys Res Commun (2021) 559:183–90. doi: 10.1016/j.bbrc.2021.04.096 33945996

